# Computational identification of strain-, species- and genus-specific proteins

**DOI:** 10.1186/1471-2105-6-279

**Published:** 2005-11-23

**Authors:** Raja Mazumder, Darren A Natale, Sudhir Murthy, Rathi Thiagarajan, Cathy H Wu

**Affiliations:** 1 Department of Biochemistry and Molecular Biology, Georgetown University Medical Center, 3900 Reservoir Rd., NW, Washington, DC 20057-1414, USA; 2 DCWASA-DWT, 5000 Overlook Ave., SW, Washington, DC 20032, USA

## Abstract

**Background:**

The identification of unique proteins at different taxonomic levels has both scientific and practical value. Strain-, species- and genus-specific proteins can provide insight into the criteria that define an organism and its relationship with close relatives. Such proteins can also serve as taxon-specific diagnostic targets.

**Description:**

A pipeline using a combination of computational and manual analyses of BLAST results was developed to identify strain-, species-, and genus-specific proteins and to catalog the closest sequenced relative for each protein in a proteome. Proteins encoded by a given strain are preliminarily considered to be unique if BLAST, using a comprehensive protein database, fails to retrieve (with an e-value better than 0.001) any protein not encoded by the query strain, species or genus (for strain-, species- and genus-specific proteins respectively), or if BLAST, using the best hit as the query (reverse BLAST), does not retrieve the initial query protein. Results are manually inspected for homology if the initial query is retrieved in the reverse BLAST but is not the best hit. Sequences unlikely to retrieve homologs using the default BLOSUM62 matrix (usually short sequences) are re-tested using the PAM30 matrix, thereby increasing the number of retrieved homologs and increasing the stringency of the search for unique proteins. The above protocol was used to examine several food- and water-borne pathogens. We find that the reverse BLAST step filters out about 22% of proteins with homologs that would otherwise be considered unique at the genus and species levels. Analysis of the annotations of unique proteins reveals that many are remnants of prophage proteins, or may be involved in virulence. The data generated from this study can be accessed and further evaluated from the CUPID (Core and Unique Protein Identification) system web site (updated semi-annually) at .

**Conclusion:**

CUPID provides a set of proteins specific to a genus, species or a strain, and identifies the most closely related organism.

## Background

Over 200 pathogenic and non-pathogenic bacteria have been completely sequenced [1], including multiple strains from several species. The availability of sequence data from related genomes has facilitated comparative genomic analysis, which not only allows the study of major evolutionary processes, but also the determination of proteins conserved across — or unique to — different species [2-4]. Information on the presence or absence of genes is a powerful tool to gain knowledge about the metabolism, pathogenicity, physiology and behavior of different organisms [2-4]. It can also provide the basis for the detection of pathogens in a given sample and to distinguish between pathogenic and non-pathogenic relatives — critical to combating disease and to the emerging biodefense field.

Two recent studies (ORFanage [5] and Procom [6]) have focused on species- and clade-specific genes, both noting a substantial number of unique genes encoded in specific organisms. The ORFanage database includes about 32,000 unique ORFs (ORFans) from 84 fully sequenced microbial genomes. An ORFan is a protein that failed to hit any other protein encoded within those 84 genomes with an E-value better than 10e^-3 ^(or 10e^-5 ^for alignments of <80 residues) using BLAST [7]. The Procom database compared proteins from thirty completely sequenced eukaryotic genomes. The proteins were pair-wise compared using WU-BLASTP [8] with a threshold E-value of 1.

We developed a general protocol to identify proteins unique to different taxa, and applied it to a set of food- and water-borne pathogens. Specifically, we: a) identify proteins that are unique to a particular strain, species, or genus; b) extract the set of proteins common to two or more strains or species; and c) determine the organism most closely related to a particular genus, species or strain. The information generated from this study is available from the CUPID (Core and Unique Protein Identification) system via a flexible and easy-to-use web interface.

## Construction and content

### Definition of terms

Throughout this paper the following terms apply:

#### Organism

The leaf-most taxonomic node indicated for a given fully-sequenced genome. The node can be at the species, strain, or sub-strain taxonomic level.

#### Self

The source strain, species or genus of a BLAST query when considering strain-, species-, and genus-specific proteins, respectively. For example, a protein encoded by any *Bacillus *is considered self at the genus level when the query organism is *Bacillus anthracis *strain Ames.

The following terms refer to the protein sets generated by this study:

#### Core

Proteins that are present in all selected organisms. "Selected organisms" will include all the completely-sequenced genomes in the data set that are taxonomically identical to the query organism at the strain, species or genus level.

#### Unique

Proteins that have no related sequences in non-self organisms. Related sequences are mined from all sources (not just from completely-sequenced genomes). Related sequences from the query organism (paralogs) are ignored as self.

#### Core unique

Proteins that have related sequences in all selected organisms, but not in non-selected organisms.

The following terms refer to the evidence provided for the indicated uniqueness status on the results page. Unless otherwise indicated, the evidence is based on BLAST results:

#### Hits

The query retrieves a protein from a non-self organism with an e-value better than 0.001.

#### Identical protein

The query sequence is identical to a sequence from a non-self organism.

#### Reciprocal hit

The best non-self hit has the original query as *its *best non-self hit. Reciprocal best hits represent an approximation of orthology [3].

#### No external hits

The query fails to hit a protein from a non-self organism using 0.001 as a cutoff.

#### No reverse hit

The best hit does not retrieve the original query when testing for reciprocal hits in a reverse BLAST. Additional evidence is provided for such cases. *Forward*gives the E-value for the hit between the query and subject. *Maxrev*gives the worst E-value reported when the subject is used as query.

#### Unclear

The best hit retrieves the original query in a reverse BLAST, but it is not the reciprocal best hit. The E-values for the *forward*and *reverse*BLASTS are given.

#### Curator judgment

"Unclear" cases that were resolved after manual checking of the data.

#### PAM30

The results shown are for BLAST using the PAM30 matrix. The default matrix is BLOSUM62.

### Data source and algorithm

**Figure 1:**
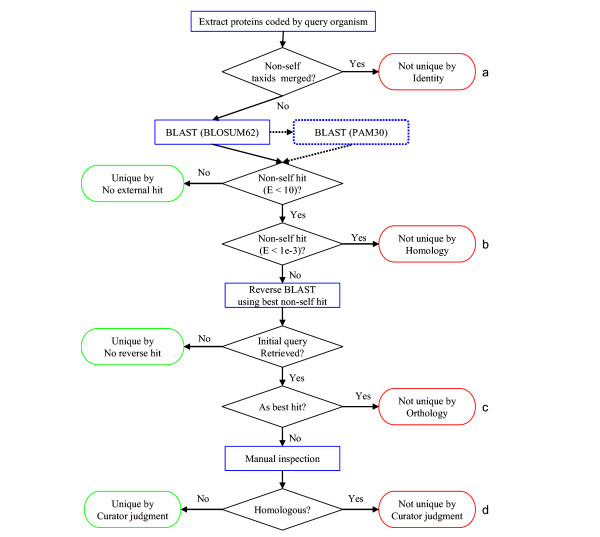
Flow chart of the method used to detect strain-, species-, or genus-specific proteins. Proteins were designated as not unique if (a) The protein is part of a merged entry (containing identical proteins from different organisms) and one of the source organisms is considered non-self; (b) The query protein hits a non-self subject with E < 0.001; (c) The non-self best hit, when itself used as a query (in a reverse BLAST), retrieves the initial query as an organism-specific best hit (that is, they are reciprocal best hits, and thus potential orthologs); and (d) The non-self best hit fails to retrieve the initial query as its potential ortholog — but does indeed retrieve the initial query — and manual inspection reveals homology. Sequences that could not retrieve themselves with an expect value of 1e^-14 ^or better in the forward BLAST using the BLOSUM62 matrix were retested using the PAM30 matrix.

Complete proteomes were retrieved from the NREF database at PIR [9], a comprehensive (~2.1 million sequences) non-redundant protein database. Identical sequences from a given species are merged in this database. Sequence searches were conducted using the BLAST program. An initial set of organism-specific proteins was generated computationally. Proteins that could not be confirmed as unique by computer analysis were manually checked by several methods [10], including multiple sequence alignment [11], hidden Markov models [12], and PSI-BLAST [7]. Proteins specific to a set of organisms were identified by determining which of the conserved core proteins [13,14] are unique (using the same procedure as used to determine organism-specific proteins). A flowchart of the process is shown in Figure 1.

Certain sequences failed to retrieve themselves using the BLOSUM62 matrix. It is known that the PAM matrices may increase the information content in the alignment and hence optimize the alignment score [15]. The PAM matrices were evaluated to identify the optimal word size, gap existence cost and gap extension cost values to be used. BLAST results using the different PAM matrices and different parameters were manually checked to identify which sequences should be analyzed using a different matrix (data not shown). We found that sequences that could not retrieve themselves with an expect value of 1e^-14 ^or better in the forward BLAST using the BLOSUM62 matrix (usually short sequences <30 aa) should be queried again using the PAM30 matrix (word size = 2, gap existence cost = 9 and gap extension cost = 1) to improve the ability to retrieve homologs. The inclusion of the reverse BLAST step and optimized search parameters both yield an analysis method that is skewed toward tagging proteins as not unique whenever possible.

The information generated for each organism at each taxonomic level (genus, species, or strain) is stored in a flat-file. Data for each protein is given on a single machine-parsable line, and is tracked using a unique protein identifier to allow integration with other PIR resources. The line contains the protein ID, an indication of whether homologs were found (+ for yes, ! for no), evidence for homology or lack thereof, identity and source organism for any core protein(s) found, and an indication of whether the query protein is part of the core. The results can be queried and browsed through the CUPID web interface. Additional analysis of the data is facilitated by links to PIR protein resources [9] and other bioinformatics servers.

### Data Update

Unique proteins for common food- and water-borne pathogens will be re-determined every six months using pre-computed BLAST (generated quarterly). *De novo *re-computation is necessary due to the constant increase in (and possible change to) protein sequences. We will add selected NIAID category pathogens with each semi-annual update cycle. In addition, we provide a mechanism to request additional proteome analyses. Users may select from a list of organisms, accessible from the web interface, that is populated by proteomes with stable sequence annotation in the UniProt Knowledgebase (UniProtKB) [16]. Analysis time will depend on several factors, including proteome size and evolutionary distance from other proteomes. The user will be notified upon completion of the analysis. Results from user-selected proteomes will be made available from the CUPID web site. Such analyses will be stamped with the date of the last computation, but will not be part of the regular update cycle.

## Usage and utility

### Retrieving strain-, species-, or genus-specific proteins

**Figure 2:**
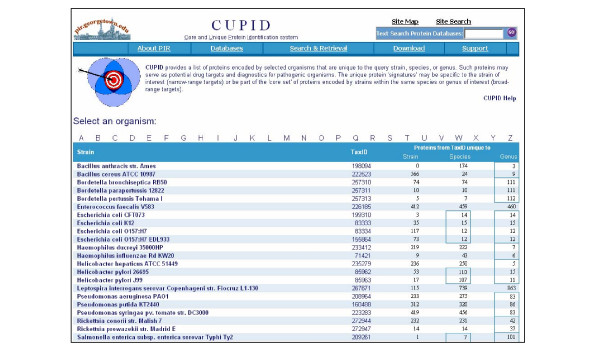
CUPID homepage. The number of core unique proteins encoded by each genome at the strain, species, and genus levels are indicated, as are the organisms considered for the core at the species and genus levels (boxes).

**Figure 3:**
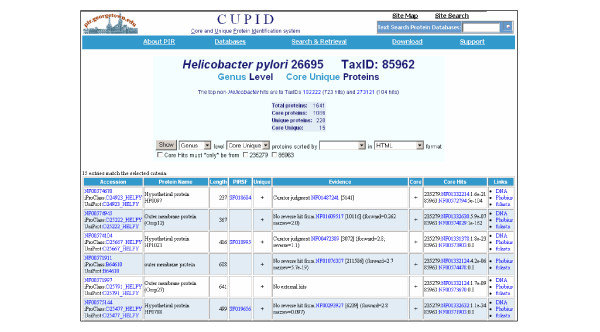
Example output from CUPID. *Helicobacter pylori *26695 was selected to retrieve proteins encoded only in bacteria of the *Helicobacter *genus. The results display protein accessions, name, length, family classification, uniqueness status, evidence for uniqueness, core status, other core proteins, and additional links. Accession links (first column) connect the protein to (i) the protein entry report, (ii) iProClass [24] entry report with rich links to over 90 molecular databases, and (iii) the UniProtKB report. Additional links (last column) are provided to (i) the EMBL DNA sequence (derived from the UniProtKB cross-reference), (ii) a pre-filled form for tBLASTn  for protein search against translated NCBI DNA databases (with Entrez post-processing set to filter out self hits) and (iii) signal sequence and transmembrane domain prediction using Phobius [25].

Unique proteins are retrieved by clicking an organism listed on the CUPID homepage (Figure 2), followed by clicking on the taxonomic level of interest. By default, the initial retrieval returns only core unique proteins (Figure 3). To retrieve just the unique proteins, one should select the taxonomic level of interest and "Unique" from the pull-down menus. For example, selecting "Genus" level "Unique" proteins for *E. coli *O157:H7 will display all proteins in *E. coli *O157:H7 that may or may not be present in other *Escherichia*, but are not present in any other genus. The "Unique" column will either have a plus sign (+), be left blank, or have a question mark (?). A plus sign means that the protein is unique by "no external hits", by no reverse BLAST hit, or by curator judgment. Blank means that the protein is not unique, and a question mark means that the relationship is of unclear status (that is, it cannot be unambiguously determined by computational means alone, and human judgment is required). The core status column indicates if the given protein has homologs in all proteomes considered self, while the core hits column lists the best hit protein from each of the self proteomes. The user can save the retrieved list in tab-delimited format or save the protein sequences in fasta format.

### Retrieving the set of proteins specific to two or more organisms

Proteins unique to two or more organisms can be retrieved from the species- or genus-specific proteins results page. This is done, in effect, by redefining the proteomes to be considered for the core. For example, once the results for *E. coli *O157:H7 are displayed, one can display the proteins unique to *E. coli *O157:H7 and *E. coli *O157:H7 EDL933 by checking the box for "Core Hits must *only* be from" and the box for Tax ID 155864. This will retrieve proteins that are conserved in both *E. coli *O157:H7 and *E. coli *O157:H7 EDL933, but are not found anywhere else. Alternatively, selecting "Show Genus level All proteins" with the same checkboxes selected as above will display all the proteins conserved between *E. coli *O157:H7 and *E. coli *O157:H7 EDL933 strains – not just the unique ones – but will exclude those found in other *Escherichia *proteomes.

### Retrieving all the proteins from a particular organism

All the proteins from an organism can be retrieved from any results page by selecting "Show XX level All proteins" (where XX can be Genus, Species, or Strain).

### Determining the closest relative

The genus, species, or strain that is most closely related to the selected organism based on the best BLAST hits of its entire proteome can be identified by clicking on the TaxID for the top non-self hits on the results page. For example, from the result page of genus-specific proteins of *E. coli *O157:H7, the top non-*Escherichia *hits are to *Shigella flexneri*, and from the result page of strain-specific proteins for *E. coli *O157:H7 the top non-*Escherichia coli *O157:H7 hits are to *E. coli *O157:H7 EDL933 (not shown).

### Description of selected results

We identified the unique proteins in thirty organisms (predominantly food- or water-borne bacterial pathogens and their close relatives). The results for ten common water and wastewater strains of *Escherichia*, *Salmonella *and *Helicobacte*r are described below. *E. coli *is the primary indicator organism for biologically contaminated food and water. Several strains of *E. coli*, such as the laboratory strain K12 [17], are completely harmless, while *E. coli *CFT073 is an extra-intestinal uropathogenic bacterium and the O157:H7 strains are enterohemorrhagic pathogens. *S. enterica *Typhi is a human pathogen that causes enteric typhoid fever. The Ty2 strain of *S. enterica *was isolated before the advent of antibiotics and has no plasmids, whereas the CT18 strain harbors a multiple-drug-resistance plasmid and a cryptic plasmid. *S. typhimurium *LT2 is a strain of *S. enterica *(synonym: *S. enterica *subsp. enterica serovar Typhimurium strain LT2). *H. pylori *is associated with peptic ulcers and several types of gastric cancer. *H. hepaticus *causes chronic hepatitis and is also a recognized carcinogen. A summary of computer-assisted manual analyses of these organisms is shown in Table 1.

**Table 1:** Strain-, species-, and genus-specific core unique proteins from selected water- and wastewater-borne pathogens and pathogen-related organisms.

Organism	Strain-specific proteins	Species-specific proteins	Genus-specific proteins
*Escherichia coli CFT073*	3	14	14
*Escherichia coli K12*	35	15	15
*Escherichia coli O157:H7*	117	12	12
*Escherichia coli O157:H7 EDL933*	73	12	12
*Salmonella enterica subsp. enterica serovar Typhi Ty2*	1	7	101
*Salmonella enterica subsp. enterica serovar Typhi str. CT18*	38	7	101
*Salmonella typhimurium LT2*	37	62	106
*Helicobacter hepaticus ATCC 51449*	236	250	5
*Helicobacter pylori 26695*	53	110	15
*Helicobacter pylori J99*	17	107	11

The number of unique proteins in each of four *E. coli *strains (K12, CFT073, O157:H7 and O157:H7 EDL933) did not show any apparent correlation with the pathogenic nature of the organism. For example, *E. coli *CFT073 (pathogenic) has only three unique proteins compared to 35 in K12 (non-pathogenic) and 117 and 73 in O157:H7 (pathogenic) and O157:H7 EDL933 (pathogenic) strains, respectively. High numbers of unique sequences in some organisms could be due to the unavailability of a closely related non-self genome or, as is likely in this example, unique plasmids that may be present in the query genome.

The number of genus/species-specific proteins for each *E. coli *is not identical as one might first expect; rather, they range from 12 to 15. This phenomenon can also be seen in other organisms where there are multiple genomes from the same species or genus. Three explanations are possible: i) each strain has different numbers of paralogs; ii) a frameshift produces two annotated open reading frames in one strain where another has one; iii) the comprehensive dataset used contains largely duplicate but non-identical entries.

The number of unique proteins for several *E. coli *strains drops as one compares strain level to genus level (except for the CFT073 strain), a trend that is also apparent for the *Helicobacter *strains. However, the opposite is true for the *Salmonella*. The progression is not necessarily linear or consistent. For example, the number of unique proteins from the CT18 strain of *Salmonella enterica *first drops from 38 at the strain level to 7 at the species level before again rising to 101 at the genus level.

A majority of the unique proteins detected in this study lacked meaningful functional annotation (i.e., were annotated simply as "hypothetical protein") (Table 2). The available annotation for the remainder indicates a preponderance of proteins with some relationship to pathogenesis or virulence (22%), or derived from phages (25%). In addition, a combination of annotation and subcellular localization prediction using PSORT-B [18] indicates that 26% of the proteins are external to the cell (cell wall attached or secreted).

**Table 2:** Summary of annotations for genus-specific unique proteins from selected water- and wastewater-borne pathogens and pathogen-related organisms.

Organism	Unique	Virulence^a^	External	Phage	Unknown	Misc.
*Escherichia coli CFT073*	182	19	28	0	134	1
*Escherichia coli K12*	262	0	8	0	254	0
*Escherichia coli O157:H7*	259	9	19	8	213	10
*Escherichia coli O157:H7 EDL933*	187	18	12	21	114	22
*Salmonella enterica subsp. enterica serovar Typhi Ty2*	244	19	24	0	198	3
*Salmonella enterica subsp. enterica serovar Typhi str. CT18*	307	4	3	19	268	13
*Salmonella typhimurium LT2*	233	0	2	43	181	7
*Helicobacter hepaticus ATCC 51449*	253	13	6	18	171	45
*Helicobacter pylori 26695*	199	9	16	5	159	10
*Helicobacter pylori J99*	663	11	1	2	637	12
**TOTAL**	**2789**	**102**	**119**	**116**	**2329**	**123**

^a^Includes proteins derived from pathogenicity islands.

## Discussion

It is evident from this study that a major reason that few unique proteins are found in some cases is the presence of sequence data for closely-related organisms and, by extension, the peculiarities of taxonomic designations. Therefore, only a general trend in the number of strain-, species-, or genus-specific proteins can be established. Strains with closely related sequenced strains tend to have relatively few unique proteins at that level while the converse is true for those without close relatives (compare *Helicobacter pylori *strains with *Helicobacter hepaticus*). The trend also holds true at the genus level (compare *Escherichia *or *Helicobacter *genus-specific proteins with *Salmonella*). We note that a proteome from a closely-related genus is represented in the protein database for both *Escherichia *(*Shigella*) and *Helicobacter *(*Campylobacter*). *Shigella *is so similar to *E. coli *that there are recommendations to consider them different species within the same genus [19], while *Helicobacter pylori *was once *Campylobacter pylori*.

The number of apparently unique genes encoded in specific organisms can depend on the definition of "unique" and the parameters and underlying database used to identify these proteins. For example, CUPID identifies 110 proteins unique to *H. pylori *26695 at the species level, whereas the ORFanage database lists 260 proteins. The ORFanage database considers the protein HP0052 to be unique. BLAST with HP0052 (UniProt ID: O24893_HELPY) retrieves several non-*Helicobacter *proteins (*Moraxella nonliquefaciens *- E = 9e^-22^, *Ehrlichia canis *- E = 3e^-18^, *Mycoplasma mycoides *- E = 2e^-15^). None of these non-self hits have a complete genome sequence deposited in the database, and therefore were not considered during the construction of the ORFanage. In addition, other lists of unique proteins consider a protein to be unique if it did not produce a BLAST hit with the E value cut-off set to 10^-3 ^[20,21], and do not further assess cases that have lower similarity. However, the contribution of low-similarity homologs is significant. For example, 950 proteins from the organisms presented in Table 1 that were considered *not *unique at the genus level were considered so because of a reciprocal best hit. Overall, we found that about 22% of proteins were removed from the list of unique proteins based on the reciprocal best hit criterion. In general, many sequences are considered to be singleton ORFs by other studies but not considered unique in this study because: a) a limited protein set was used for BLAST comparison as opposed to a comprehensive database; b) the definition of *self *is different (strain differences may not be considered); c) only unidirectional BLAST hits (without reverse BLAST) are considered to find homologs; and d) the cutoff used to define homolog is less conservative. Using a thorough computational method and a comprehensive database leads to a more conservative estimate of the number of unique proteins.

Despite the conservative approach used here, one must be mindful of certain pitfalls in deriving lists of unique proteins. First, a protein might be labeled as unique only because homologs from other organisms were missed upon submission of the sequence, or because of some other conceptual translation problem. In all cases, the short list of potential unique proteins should be further screened computationally at the DNA level using tBLASTn. Running tBLASTn using a protein of interest will make sure that the gene is indeed unique, at least with respect to the current pool of submitted sequences (to aid in this task, we have included a link to NCBI's tBLASTn web page). Second, many of the proteins identified here are remnants of prophage proteins [22]. The leading role of bacteriophages in shaping the *E. coli *O157:H7 genome is evident by the presence of 24 prophages and prophage-like elements, and several genes that have been laterally transferred [23]. At the strain level, labeling of such proteins as unique may be more a reflection of a gap in whole-genome sequence information than of true specificity. Accordingly, discrimination between individual strains (isolates) may require laboratory comparison methods such as pulsed-field gel electrophoresis or whole-cell fatty acid analysis. In contrast, identifying species- or genus-specific proteins can be done with confidence when multiple representatives have been sequenced. In such cases, conservation within multiple strains of a species (for example) gives confidence in the "reality" of the uniqueness because that status has been conserved over time (core unique proteins).

Precise identification of pathogens is important so that adequate action can be taken to either eliminate or reduce the threat of infection. One use of the CUPID system is to help identify diagnostic targets specific to a particular clade of these pathogens. The unique proteins form a short list of diagnostic targets to be validated in the laboratory. Proteins predicted to be external to the bacterial cell – possibly involved in host interactions and virulence – may be used to develop protein-based detection systems. In addition, it should be possible to use the DNA encoding these proteins as the basis for diagnostics. However, it is important to note that protein-identified DNA probes must be verified as to their uniqueness at the DNA level.

## Conclusion

The salient features of CUPID are: a) provides sets of proteins unique to a strain, species, and genus level; b) includes a check for additional homologs based on reciprocal hits; c) uses different parameters for short sequences; d) provides the identity of the nearest non-self neighbor; and e) allows retrieval of unique, core, and core unique proteins at different taxonomic levels.

## Availability and requirements

CUPID is freely accessible from the PIR website at .

## List of abbreviations

CUPID – Core and Unique Protein Identification system

PIR – Protein Information Resource

## Authors' contributions

RM conceived, designed and coordinated the study, developed a general outline for the algorithm and drafted the manuscript. DN developed the specific algorithm and was responsible for software design and implementation, and participated in the writing of the manuscript. CW and SM participated in the design and evaluation of the study and manuscript writing. RT and DN developed the web interface. All authors read and approved the final manuscript.
